# Enzyme-Triggered Formation of Tensegrity Structures for Mechanospatial Manipulation of Hydrogels

**DOI:** 10.3390/gels11080654

**Published:** 2025-08-18

**Authors:** Juan Wang, Xu Han, Qingtai Li, Meng Qin, Bin Xue, Wenxu Sun, Yi Cao, Wei Sun

**Affiliations:** 1 Collaborative Innovation Center of Advanced Microstructures, National Laboratory of Solid State Microstructure, Department of Physics, Nanjing University, Nanjing 210008, China; wangjuannm@163.com (J.W.); dz20220007@smail.nju.edu.cn (X.H.); lqt861563391@163.com (Q.L.); qinmeng@nju.edu.cn (M.Q.); xuebinnju@nju.edu.cn (B.X.); caoyi@nju.edu.cn (Y.C.); 2 Jinan Microecological Biomedicine Shandong Laboratory, Jinan 250000, China; 3 School of Physical Science and Technology, Nantong University, Nantong 226019, China; 4 Chemistry and Biomedicine Innovation Center, School of Chemistry and Chemical Engineering, Nanjing University, Nanjing 210000, China

**Keywords:** hydrogel, mechanical properties, tyrosine crystals, biomaterials

## Abstract

Hydrogels with spatially programmable mechanical properties hold great potential for use in biomedical applications. Inspired by the architecture of the cytoskeleton, we present a strategy for constructing tensegrity-structured hydrogels (TS-Gels) through enzyme-triggered crystal growth to enable precise mechanospatial manipulation. Specifically, alkaline phosphatase (ALP) was covalently anchored to a polyacrylamide (PAAm) hydrogel matrix to catalyze the in situ dephosphorylation of phosphotyrosine precursors, leading to the formation of rigid tyrosine crystals. These crystals functioned as compressive sticks, establishing tensegrity structures within the hydrogel network. By tuning the crystallization kinetics, both the structural morphology and mechanical reinforcement could be precisely controlled. The resulting TS-Gels exhibited significantly enhanced local tensile strength and stiffness, allowing for spatial–mechanical patterning via photo-initiated printing, mold-assisted shaping, and laser engraving. Furthermore, the unique mechanospatial tunability of TS-Gels was demonstrated in tribological surface engineering, underscoring their potential for use in tissue engineering and responsive biomaterials.

## 1. Introduction

Hydrogels with spatially tunable mechanical properties can mimic the native microenvironment of biological tissues and are therefore promising materials for use in biomedical engineering [1,2]. For instance, hydrogels with modulus gradients have shown outstanding performance in load-bearing and low-friction biological lubrication, effectively prolonging the service life of motion-related implants such as artificial joints [3,4,5]. Moreover, mechanically programmable scaffolds with spatial stiffness control can direct stem cell differentiation along specific lineages, significantly enhancing the tissue regeneration efficiency [6,7]. These advances highlight the potential of hydrogels for use as functional interfaces in complex biological systems. However, achieving precise mechanospatial control, where the local stiffness and structural organization can be programmed on demand, remains a fundamental challenge that limits their broader biomedical and engineering applications [8].

The current strategies for mechanical manipulation of hydrogels—such as double-network design [9,10,11,12], mechanochemical coupling [13,14,15], polymer chain entanglement [16,17], and hidden length release [18,19]—have successfully enhanced the global stiffness and toughness. Fabrication techniques including self-assembly [20], directional templated growth [21,22], and flow-assisted assembly [23], can generate mechanical anisotropy at the nano- or microscale. However, these approaches generally target either bulk property enhancement or micro/nanoscale anisotropy and thus cannot prescribe mechanical properties at the millimeter scale with a high spatial resolution.

The main barriers to mechanospatial control of hydrogels arise from three key factors. First, suitable chemical reactions for localized stiffness tuning must be both biocompatible and spatially addressable; however, many existing chemistries lack this level of controllability [24,25]. Second, fabrication methods often rely on a uniform precursor distribution or global post-processing, making it difficult to selectively pattern mechanical domains without compromising the surrounding regions [26,27]. Third, integrating anisotropic reinforcement architectures in situ while maintaining precise topological organization across length scales remains technically challenging [28,29].

To address these challenges, enzyme-triggered biomineralization offers a promising approach for locally modulating the stiffness over a broad range (0.1–100 MPa) [30,31]. Drawing inspiration from biological structures such as the cytoskeleton [32,33,34], extracellular matrix [35,36], and connective tissues [37], enzyme-triggered formation of tensegrity structures emerges as a compelling strategy for mechanospatial programming in hydrogels. These architectures consist of rigid, compression-bearing sticks interwoven with a continuous tension network [38,39,40]. While our previous work demonstrated that incorporating tensegrity-like elements enhances the bulk modulus and toughness of hydrogels [41,42], their typically random distribution limits their usefulness in applications requiring spatially defined mechanical features and thus hinders their utility in precise mechanospatial programming. This limitation arises primarily because controlling the biomineralization kinetics and crystal topology for forming organized tensegrity structures within complex hydrogel environments remains difficult.

Here, we report a strategy to construct spatially programmable tensegrity structures within hydrogels. Tensegrity structures are characterized by a stable balance between discontinuous compressive elements and a continuous tensile network, enabling efficient force distribution and mechanical resilience. By covalently anchoring alkaline phosphatase (ALP) enzymes to a polyacrylamide (PAAm) hydrogel network, in situ dephosphorylation of phosphotyrosine precursors induces the site-specific growth of tyrosine crystals, which act as rigid compressive sticks. Combined with the prestressed PAAm matrix acting as a continuous tensile network, this leads to the formation of tensegrity-structured hydrogels (TS-Gels) with tunable local stiffness ranging from 0.05 to 25 MPa. Spatial–mechanical patterning is readily achieved through light-initiated printing, molding, or laser engraving. This approach offers a simple yet powerful method for the mechanospatial manipulation of hydrogels, with promising applications in tribological surface engineering and biomaterial applications.

## 2. Results and Discussion

### 2.1. Design and Characterization of TS-Gel

As a representative tensegrity structure in biological systems, the red blood cell cytoskeleton is formed through the hierarchical assembly of actin filaments and microtubules, providing mechanical stability and facilitating cell motility [43,44,45]. In this structure, microtubules act as interlocking rigid elements, while tensile actin filaments serve as soft connectors. This arrangement constructs an elastic framework that efficiently responds to external mechanical stress (Supplementary Figure S1, top), enabling cells to resist deformation as they pass through complex capillary networks and inter-endothelial junctions.

**Figure 1:**
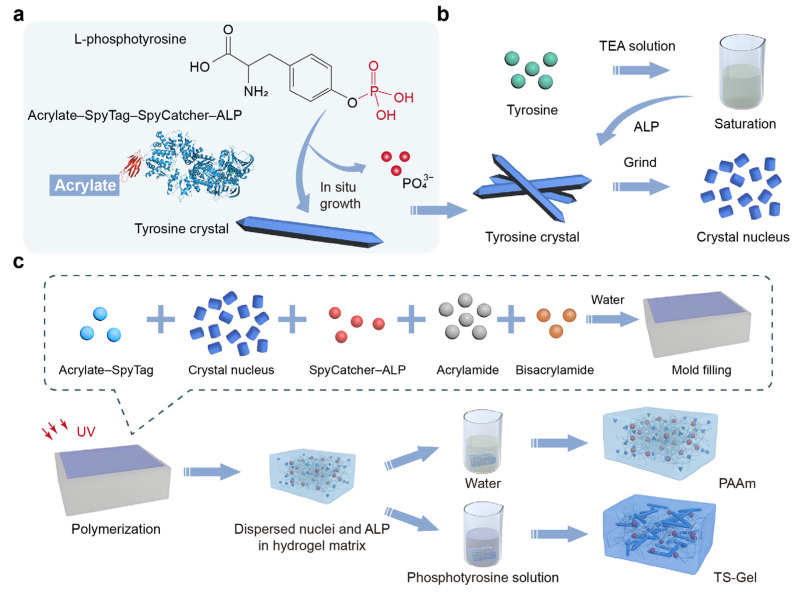
Strategy for regulating the local stiffness in hydrogels using in situ growth of tyrosine crystals to form tensegrity structures. (**a**) Schematic of the in situ growth of tyrosine crystal sticks within the hydrogel network, catalyzed by alkaline phosphatase (ALP). (**b**) Preparation of crystal sticks and nuclei. (**c**) Formation of tensegrity structures in the hydrogel through ALP-induced in situ crystallization. ALP is covalently incorporated into the hydrogel matrix to trigger localized crystal growth. The spatial–mechanical properties of the hydrogel can be precisely tuned by controlling the crystal growth time, the distribution of the nucleation sites, and the ALP concentration.

Emulating these tensegrity structures, our hydrogels were composed of a prestressed polymer network and mechanically interlocked amino acid crystals (Supplementary Figure S1, bottom). The key to achieving mechanospatial manipulation lay in the in situ generation of prestressed tyrosine crystals (Figure 1a). This process began with the covalent attachment of alkaline phosphatase (ALP) to the hydrogel network, which triggered the dephosphorylation and subsequent growth of amino acids. As these crystals elongated, they stretched the surrounding polymer network. The micron-sized crystals thus exerted tensile stress on the soft polymer network, which in turn applied compressive stress to the crystals. This interaction formed a cytoskeleton-like structure, where the amino acid crystals functioned as rigid elements and the components of the prestressed polymer network served as soft connectors.

To form the matrix of the TS-Gel, a single polyacrylamide (PAAm) network crosslinked with bisacrylamide was employed. Alkaline phosphatase (ALP) was covalently attached to the hydrogel network using a genetically engineered SpyCatcher–ALP chimera via SpyCatcher–SpyTag chemistry [46]. Micron-sized tyrosine crystal seeds were incorporated as nucleation sites into the precursor solution of the PAAm hydrogels (Figure 1b, Supplementary Figure S2). The precursor mixture, comprising acrylamide, bisacrylamide, acrylate–SpyTag, SpyCatcher–ALP, and the crystal seeds, was gelled in a single step. Following dialysis in a triethylamine (TEA) buffer (0.2 M, pH = 8.8) for 6 h, the hydrogel containing the crystal seeds was incubated in a saturated L-tyrosine solution supplemented with 50 mM phosphotyrosine for 120 h to facilitate crystal growth and form the TS-Gel (Figure 1c, Supplementary Figure S3). Control PAAm hydrogels were prepared using an identical procedure to that for the TS-Gel, but without the subsequent crystal growth. Fourier transform infrared spectroscopy (FTIR) and X-ray diffraction (XRD) analyses confirmed the successful growth of tyrosine crystals within the TS-Gel (Supplementary Figure S4). Furthermore, thermogravimetric analysis (TGA) and differential thermal analysis (DTA) revealed that the TS-Gel exhibited higher thermal stability compared to PAAm (Supplementary Figure S5), which can be attributed to the presence of tyrosine crystals.

Tyrosine crystal sticks formed in a solution environment mimicking the hydrogel matrix exhibited a prismatic morphology with a diameter of approximately 2 μm, as revealed by atomic force microscopy (AFM) imaging (Figure S6a). AFM-based nanoindentation measurements (Figure S6b,c) showed that the crystal sticks possessed a high modulus of ~15.3 GPa, indicating their excellent compressive stiffness and suitability as structural reinforcement elements. The native PAAm hydrogel was translucent and soft, visibly sagging when held with tweezers (Supplementary Figure S7a,b). Scanning electron microscopy (SEM) revealed a porous internal microstructure characteristic of typical PAAm hydrogels (Supplementary Figure S7c). In contrast, the TS-Gel formed after growing the crystals (t = 120 h) appeared opaque (Supplementary Figure S7d) and exhibited significantly higher stiffness (Supplementary Figure S7e), which was likely attributable to the formation of tyrosine crystal sticks within the network. SEM imaging further revealed an architecture with interlocking between the crystal sticks and the surrounding polymer chains in the TS-Gel (Supplementary Figure S7c,f), confirming that the network was prestressed and under tensile strain.

Mechanical testing demonstrated that the TS-Gel formed after growing the crystals (t = 120 h) possessed enhanced tensile and compressive fracture strengths compared to conventional PAAm hydrogels, owing to the embedded tensegrity architecture. Under tensile loading, the TS-Gel displayed significantly improved mechanical properties, as evidenced by its typical stress–strain curve (Supplementary Figure S7g), including a high tensile modulus (~25 MPa) and a fracture strength of approximately 1.0 MPa. In contrast, under compressive loading, the TS-Gel exhibited a markedly lower compressive modulus (Supplementary Figure S7h), with a tensile-to-compressive-modulus ratio exceeding 10 (Supplementary Figure S7i). This pronounced mechanical anisotropy—referred to as dual-modulus behavior—is a hallmark of tensegrity-structured materials [47], arising from distinct modes of force transmission under tensile and compressive stress.

### 2.2. Formation and Kinetics of Tensegrity Structures

By tracking the evolution of the crystal growth, the TS-Gel provided a great opportunity to study the formation and kinetics of tensegrity structures. As the crystals grew, the hydrogel transformed from a translucent, soft state to an opaque and rigid one (Supplementary Figure S8). Scanning electron microscopy (SEM) images of the TS-Gel at 0, 0.5, and 24 h illustrate the dynamic process of crystal emergence and growth and the concurrent evolution of the network structure. After 0.5 h of growth, nascent rod-like crystals with lengths of below 5 μm began to appear in the ALP-incorporated PAAm hydrogels. These crystals subsequently elongated to over 20 μm. After 24 h of growth, nearly all the crystals exhibited a rod-like morphology with stabilized lengths of approximately 20 μm. The plateau in the increase in the crystal length after 24 h likely arose from steric and mechanical confinement within the hydrogel network, which restricted further elongation once the local polymer deformation reached a certain threshold. Prolonged incubation mainly increased the number of crystals, suggesting continued nucleation in unoccupied regions. Thus, while the local stiffness was constrained by the length limit for individual crystals, the overall reinforcement could be tuned by controlling the nucleation density.

**Figure 2:**
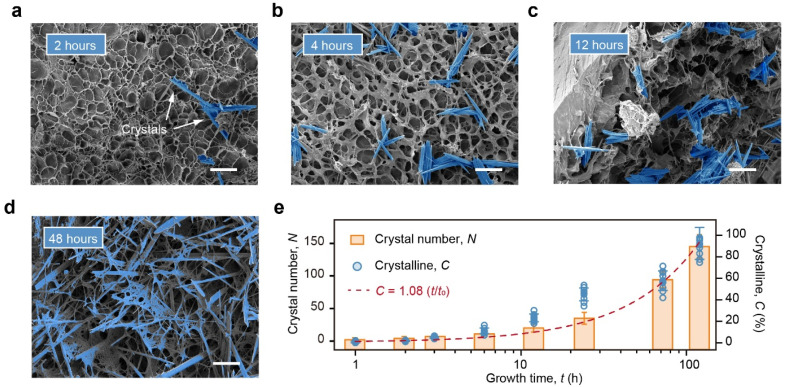
Growth kinetics of tyrosine crystals in TS-Gels. (**a**–**d**) SEM images of TS-Gels after (**a**) 2, (**b**) 4, (**c**) 12, and (**d**) 24 h of in situ tyrosine crystal growth. Scale bars: 50 μm. (**e**) Linear fitting of tyrosine crystallization as function of growth time. Blue highlighted regions indicate crystal features identified using image analysis algorithms. Crystallinity at time of 0 h is 0 and cannot be plotted due to logarithmic scale limitations. Red dashed line represents linearly fitted crystallization kinetics curve.

Tyrosine crystals within the SEM images were analyzed using a combination of image thresholding, edge-enhanced visual identification, and fiber feature recognition algorithms (Supplementary Table S2 and Supplementary Figure S9). For each 420 μm × 320 μm SEM image, the number of individual crystals was counted to evaluate the temporal evolution of crystallization. Representative SEM images of the TS-Gel samples after 2, 4, 12, and 48 h of growth were processed and highlighted (Figure 2a–d), demonstrating a clear increase in the crystal count with an increasing growth time. SEM images of the TS-Gels corresponding to growth times from 0 to 120 h were analyzed using this image processing approach. Each data point in Figure 2e represents the mean crystal number calculated from three independent SEM images per time point. At both 0 and 1 h, the number of crystals (*N*) was zero, as the incubation time was too short for detectable crystal formation. From 2 to 120 h, *N* increased gradually to 143, indicating continuous growth and accumulation of tyrosine crystals within the TS-Gel during the 120 h incubation period.

To quantify the tyrosine crystal content in the TS-Gels, the samples were weighed after incubation for different durations. The crystallinity (*C*) was calculated using the equation *C* = (*W*_t_/*W*_120_) × 100%, where *W*_t_ corresponds to the mass of the crystals in the hydrogels after incubation for t hours, and *W*_120_ corresponds to the mass of the crystals in the hydrogels after 120 h of incubation. The crystallization kinetics curve was constructed by plotting *C* versus the time (*t*). The calculated crystallinity exhibited a linear relationship with the growth time: *C* = 1.08 (*t*/*t*_120_), with an *R*^2^ value of 0.95, as shown in Figure 2e, where *t*_120_ is defined as 120 h. These results demonstrate that the extent of crystallization in tensegrity structures can be precisely tuned by controlling the growth time, providing a reliable strategy to regulate the spatial–mechanical properties of the hydrogel.

### 2.3. Spatial Programming of Tensegrity Structures

Building upon our understanding of the formation and growth kinetics of tensegrity structures in the TS-Gels, we further explored their spatial programmability by engineering graded distributions of tyrosine crystal nucleation sites. To this end, tyrosine crystal nuclei were embedded in ALP-incorporating PAAm hydrogels at varying concentrations: 0.0%, 0.1%, 0.2%, and 0.4%. These precursor hydrogels were then incubated in a phosphotyrosine solution for 24 h under identical conditions to induce in situ crystallization.

**Figure 3:**
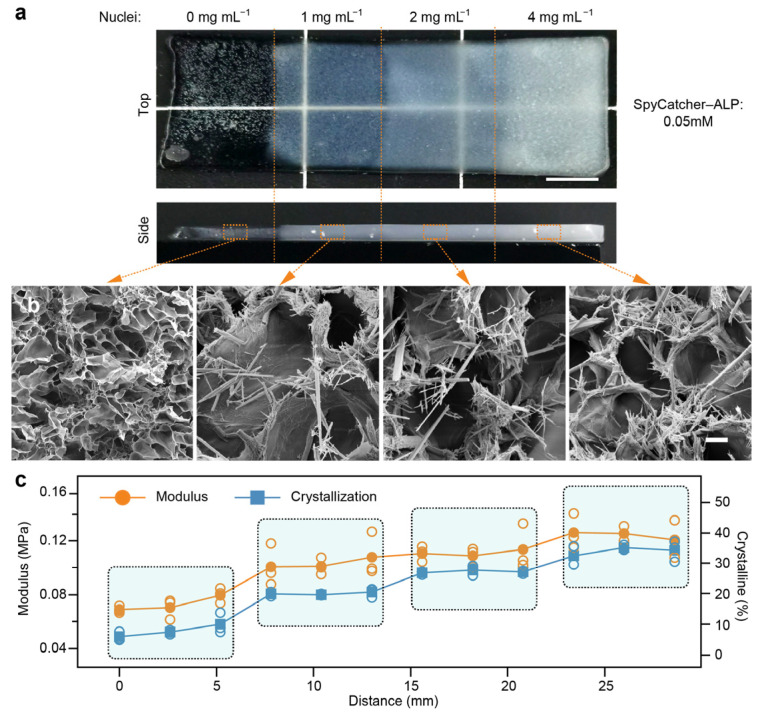
Spatial control of tensegrity structures in TS-Gels using gradient of nucleation densities. (**a**) Top-view optical images and (**b**) corresponding SEM images of TS-Gels with graded tyrosine nucleus concentrations after 24 h incubation. Scale bars: 3 mm (optical) and 100 μm (SEM). (**c**) Corresponding compressive modulus and crystallinity profiles along gradient axis. Note: x-axis labeled “Distance (mm)” represents four distinct, discrete regions rather than continuous spatial measurement. Data are presented as mean ± standard deviation (*n* = 3).

As shown in Figure 3a, a frontal optical image of the resulting gradient TS-Gel revealed four distinct regions with progressively increasing opacity, indicative of a higher crystal content. Side-view imaging further showed that whitening in the 0.0% region was largely surface-localized, whereas in the 0.4% region, the opacity extended uniformly throughout the hydrogel, suggesting more extensive crystal growth. SEM imaging confirmed the distribution of tyrosine crystal sticks in each region. In the 0.0% nuclei region, few to no crystals were observed (Figure 3b), while regions with higher nucleation densities (0.1–0.4%) exhibited increasingly dense arrays of elongated tyrosine crystal sticks. This positive correlation between the nucleation density and crystalline morphology confirmed the feasibility of spatially directing crystal formation using patterned seed concentrations.

To assess the mechanical consequences of this crystal gradient, uniaxial tensile tests were performed on 12 equally spaced zones along the gradient axis (Supplementary Figure S10). The elastic modulus exhibited a clear increasing trend, rising from 0.06 MPa in the 0.0% zone to 0.12 MPa in the 0.4% zone (Figure 3c), reflecting the reinforcement provided by the crystalline tensegrity structures. Furthermore, using weight-based quantification, we evaluated the local crystallinity across the gradient TS-Gel. These results showed a strong positive correlation between the elastic modulus and crystalline content, validating that spatially pre-patterned nucleation can effectively direct the mechanospatial properties of TS-Gels.

**Figure 4:**
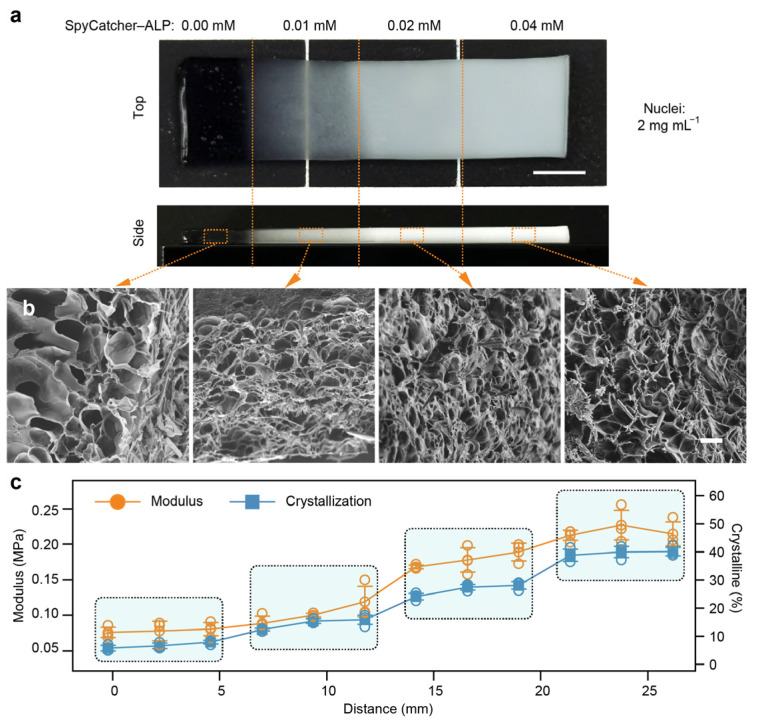
Spatial control of tensegrity structures in TS-Gels using gradient of SpyCatcher–ALP concentrations. (**a**) Top-view optical images and (**b**) corresponding SEM images of TS-Gels containing gradient of SpyCatcher–ALP after 24 h incubation. Scale bars: 3 mm (optical) and 100 μm (SEM). (**c**) Compressive modulus and crystallinity profiles measured along gradient axis. Note: x-axis labeled “Distance (mm)” represents four distinct, discrete regions rather than continuous spatial measurement. Data are presented as mean ± standard deviation (*n* = 3).

In addition to preprogrammed nucleation distributions, we also investigated the spatial programming of tensegrity structures through modulation of the ALP concentration. Precursor PAAm hydrogels containing a uniform 0.4% concentration of tyrosine crystal nuclei were supplemented with ALP at concentrations of 0.0%, 0.1%, 0.2%, and 0.4%, respectively. These precursor gels were polymerized into four distinct regions (6 mm × 6 mm), arranged in ascending order of the ALP concentration, as shown in Figure 4a. The composite hydrogels were then incubated in a phosphotyrosine solution for 24 h under identical crystallization conditions.

A top-view optical image of the ALP gradient TS-Gel exhibited four distinguishable zones with increasing opacity, consistent with the rising crystal content (Figure 4a). Side-view images revealed that the 0.0% ALP region remained translucent, while the 0.4% ALP region exhibited uniform whitening throughout the hydrogel volume, indicating pervasive crystal formation. SEM analysis confirmed this trend: no crystals were detected in the 0.0% ALP region (Figure 4b), whereas regions with higher ALP concentrations displayed progressively more abundant and elongated tyrosine crystal sticks. These results highlight the ability to spatially control crystallization through a graded ALP distribution. To evaluate the mechanical impact of the ALP gradient, uniaxial tensile tests were conducted at 12 evenly spaced positions along the gradient direction (Supplementary Figure S11). The elastic modulus increased markedly from 0.07 MPa in the 0.0% region to 0.21 MPa in the 0.4% region (Figure 4c), indicating substantial reinforcement of the hydrogel network due to the formation of tensegrity structures. Additionally, crystallinity measurements revealed a strong positive correlation between the local modulus and crystal content, providing further evidence that spatial variations in the ALP concentration can effectively program the mechanical heterogeneity of TS-Gels via enzyme-guided crystallization.

### 2.4. Patterning TS-Gels via Mechanospatial Manipulation

**Figure 5:**
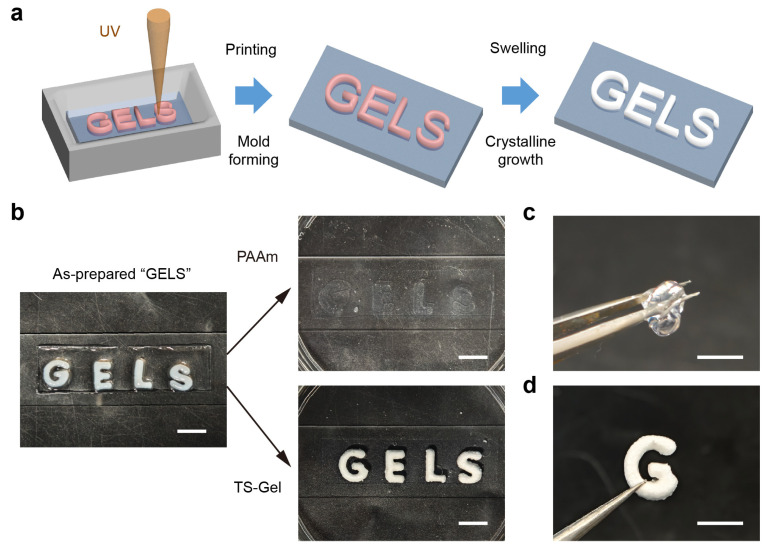
Fabrication of patterned tensegrity-structured hydrogels using light-initiated printing and mold casting. (**a**) Schematic illustration of the light-initiated printing process. A hydrogel precursor containing SpyCatcher–ALP was used to print the pattern “GELS” under UV exposure, followed by filling with an ALP-free precursor to complete the mold. The composite hydrogel underwent in situ crystal growth. (**b**) Optical image of the patterned TS-Gel showing crystal formation exclusively in the printed region. Scale bar = 3 mm. (**c**) Physical deformation of a “G”-shaped PAAm hydrogel under gravity. (**d**) A corresponding TS-Gel sample showing shape retention due to enhanced stiffness.

To enable spatially programmable formation of tensegrity structures within the TS-Gels, we implemented two complementary patterning strategies: light-initiated printing and laser engraving. Both approaches allowed for precise spatial control over the crystal growth, thereby tailoring the local crystallinity and mechanical properties within the hydrogel matrix. The light-initiated printing strategy harnessed the site-specific crystallization of tyrosine guided by localized enzymatic activity to embed mechanically distinct regions within a continuous hydrogel network. As illustrated in Figure 5a, a patterned hydrogel was fabricated by selectively printing the word “GELS” using a PAAm precursor solution containing SpyCatcher–ALP, followed by filling the remaining space with an ALP-free precursor. Upon incubation in a tyrosine phosphate solution for 120 h, crystal growth occurred exclusively in the ALP-rich regions, producing embedded crystalline domains with increased local stiffness.

The resulting spatial pattern was visually confirmed by optical imaging (Figure 5b), where the “GELS” region appeared markedly white due to dense crystal formation, in contrast to the translucent, crystal-free background. Control experiments using ALP-free precursors showed no whitening under identical conditions, confirming the enzymatic specificity of the crystallization process. Mechanical differentiation was further demonstrated via physical manipulation. As shown in Figure 5c,d, a “G” segment composed of the standard PAAm hydrogel collapsed under gravity when lifted, while its TS-Gel counterpart retained its structure and rigidity. These results underscore the utility of light-guided mechanospatial modulation in encoding the localized stiffness and architecture, offering a straightforward strategy for fabricating multifunctional, mechanically heterogeneous soft materials.

**Figure 6:**
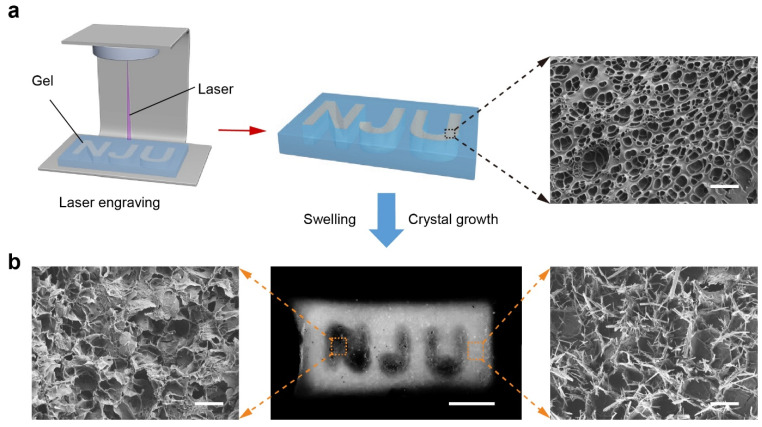
Fabrication of patterned TS-Gels via laser engraving. (**a**) Schematic of the laser engraving process. A uniform TS-Gel containing tyrosine nuclei and SpyCatcher–ALP was selectively ablated with a high-energy laser to melt nucleation sites in the shape of “NJU,” followed by in situ crystal growth. (**b**) Optical (top) and SEM (bottom) images of the patterned TS-Gel. The laser-engraved regions remained translucent and free of crystals, while untreated areas developed dense crystal networks. Scale bars = 3 mm (optical), 100 μm (SEM).

In contrast, the laser engraving strategy provided a post-fabrication method for patterning. A homogeneous TS-Gel precursor containing tyrosine nuclei and SpyCatcher–ALP was first prepared. A high-energy laser was then used to selectively ablate the crystal nuclei, engraving the pattern “NJU” (Figure 6a). Localized heating from the laser effectively destroyed the nucleation sites in the irradiated regions without compromising the overall gel structure. Subsequent incubation in the crystal growth solution induced selective crystallization only in the non-ablated regions. Optical imaging revealed that the laser-treated regions remained translucent and mechanically soft, whereas the surrounding areas became opaque and stiffer due to crystalline reinforcement. SEM analysis (Figure 6b) confirmed these observations: no crystals were observed in the ablated zones, while dense, rod-like tyrosine crystals were evident in the untreated regions. Compared to light-initiated printing, laser engraving enables flexible post-patterning of hydrogels, including the fabrication of negative-space architectures. The former supports integration of complex three-dimensional structures during fabrication, while the latter offers a high spatial resolution for modifying already-formed gels [48,49].

In summary, these spatial patterning techniques demonstrate the capacity of TS-Gels to exhibit programmable microscale mechanical anisotropy within a continuous matrix. Such control over the stiffness distribution holds great promise for mimicking the hierarchical architecture of biological tissues, such as the gradient transitions in osteochondral or ligament interfaces [50]. Moreover, it offers a versatile platform for creating integrated functional systems, including patterned soft actuators and strain-responsive sensors [51].

### 2.5. Construction and Tribological Properties of Bilayer TS-Gels

**Figure 7:**
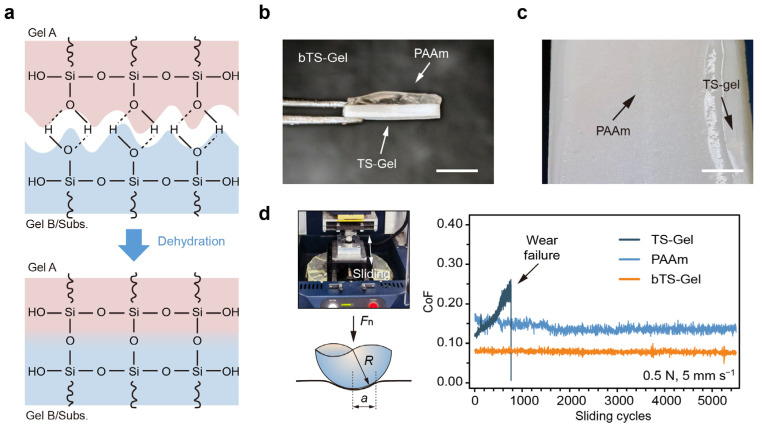
Construction strategy and tribological performance of bilayer TS-Gels (bTS-Gels) with integrated soft–stiff architecture. (**a**) Schematic of silane coupling for bonding at hydrogel–hydrogel or hydrogel–substrate interfaces. (**b**) Side-view and (**c**) top-view optical images of bTS-Gels composed of soft PAAm top layer and stiff TS-Gel bottom layer. Scale bars: 3 mm (**b**), 1 mm (**c**). (**d**) Schematic of tribological test setup and measured CoFs for PAAm, TS-Gel, and bTS-Gel over 5000 cycles.

Building upon the light-initiated printing method, we next constructed bilayer tensegrity-structured hydrogels (bTS-Gels) with vertically stratified mechanical architectures. Specifically, a composite structure was engineered by integrating a crystal-free, lubricating PAAm layer with a stiff TS-Gel base, aiming to explore how such directional mechanical asymmetry influences the tribological behavior of load-bearing hydrogel systems. To fabricate these bTS-Gels, we introduced 1.0 mM 3-(trimethoxysilyl)propyl methacrylate (TMSMMA) into both the PAAm and TS-Gel precursor solutions to enhance interfacial adhesion. The TMSMMA provided Si–OH groups capable of forming covalent bonds at the hydrogel–hydrogel and hydrogel–substrate interfaces (Figure 7a), as previously reported [52,53]. Using this approach, bilayer hydrogels with well-defined architectures were successfully fabricated (Figure 7b,c).

The coefficients of friction (CoFs) of the PAAm, TS-Gel, and bTS-Gel samples were measured using a UMT tribometer under a 0.5 N normal load and a sliding speed of 5 mm s^−1^ (Figure 7d). The PAAm hydrogel initially exhibited a low CoF (~0.12), which gradually increased due to surface degradation, ultimately leading to mechanical failure. In contrast, the TS-Gels maintained a stable CoF (~0.16) over 5000 reciprocating cycles, albeit a higher one than that of PAAm, likely due to surface roughness introduced by the embedded tyrosine crystals. Remarkably, the bTS-Gel leveraged the mechanical durability of the TS-Gel layer and the lubricity of the PAAm layer, resulting in superior tribological performance. Its CoF remained both low and stable (~0.08) throughout the test, demonstrating the effectiveness of vertical mechanical stratification in reducing friction and wear. These results highlight the potential of bTS-Gels for use in biomedical applications such as articular cartilage repair, where both wear resistance and lubrication are critical.

**Figure 8:**
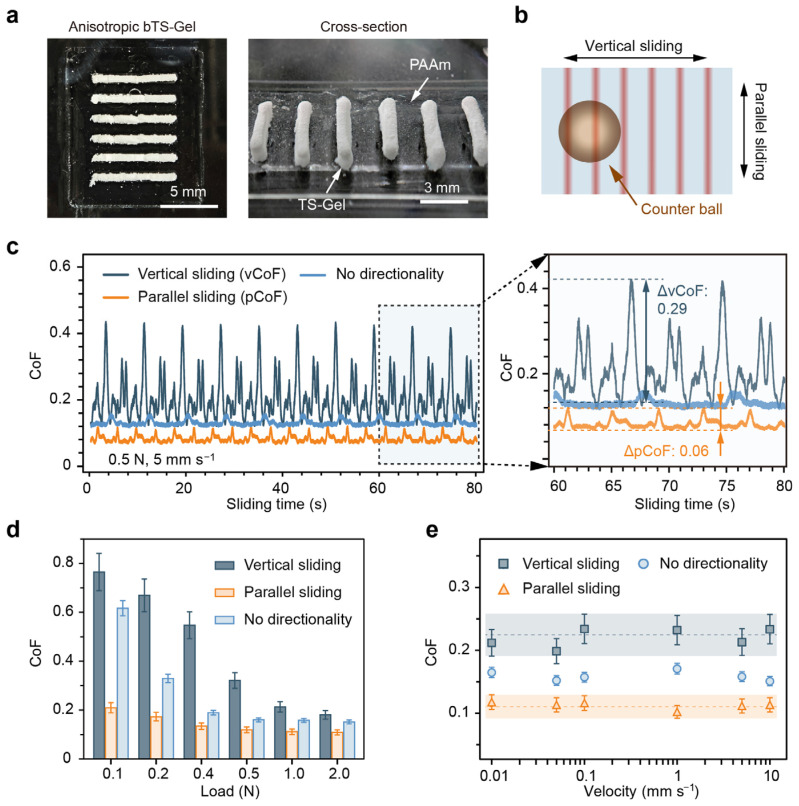
Tribological performance of anisotropic bTS-Gels. (**a**) Top and side optical images of bTS-Gels with aligned TS-Gel stick structures. White strips: TS-Gel; translucent: PAAm. (**b**) Schematic of sliding directions: parallel and perpendicular to TS-Gel strips. (**c**) CoFs for vertical and parallel directions at 0.5 N and 5 mm s^−1^. (**d**) Load-dependent CoFs (vCoF, pCoF, and control) from 0.1 to 2.0 N. (**e**) CoFs as function of sliding velocity under 0.5 N normal load.

Previous studies have shown that anisotropies in the mechanical or surface roughness of water-lubricated materials can significantly impact their tribological performance. To further explore this, we fabricated directionally anisotropic bTS-Gels using a mold-assisted strategy. The resulting hydrogels exhibited aligned tensile–stick structures, as evidenced in the optical images in Figure 8a. In the top view, the whitish strips represent the TS-Gel domains, while the translucent areas correspond to PAAm. The side view reveals the local height variations induced by the TS-Gel sticks, creating surface anisotropies in both the mechanics and roughness.

To assess the tribological behavior, we performed friction tests in two orientations—parallel and perpendicular to the TS-Gel strips—under normal loads of 0.1–2.0 N and sliding speeds of 0.01–10 mm s^−1^ (Figure 8b). Isotropic PAAm hydrogels were used as a non-directional control. Under a representative load of 1.0 N and velocity of 5 mm s^−1^, the vertically sliding samples (vCoF) displayed strong periodic fluctuations (average of ~0.213, ranging from 0.13 to 0.42), as shown in Figure 8c. In contrast, both the parallel (pCoF) and non-directional samples exhibited lower and more stable CoFs of ~0.112 and ~0.158, respectively. Notably, the pCoF showed significantly lower CoF fluctuations (ΔCoF) compared to the vCoF, indicating that alignment between the sliding direction and the tensegrity architecture enhances the lubrication stability and reduces friction.

Load-dependent testing (Figure 8d) revealed a decreasing trend in the vCoF and the control CoF with an increasing load, consistent with soft polymer gel friction behavior [54]. In contrast, the pCoF remained stable (~0.12) across the 0.1–2.0 N range, likely due to the increased stiffness and aligned stick geometry of the TS-Gel, which better conformed to Amontons′ law of friction. Additionally, Figure 8e shows that the CoF remained nearly constant across a broad range of sliding velocities, further supporting the existence of a hydration-mediated lubrication mechanism [55,56].

### 2.6. Cytocompatibility and Potential Applications of TS-Gels

**Figure 9:**
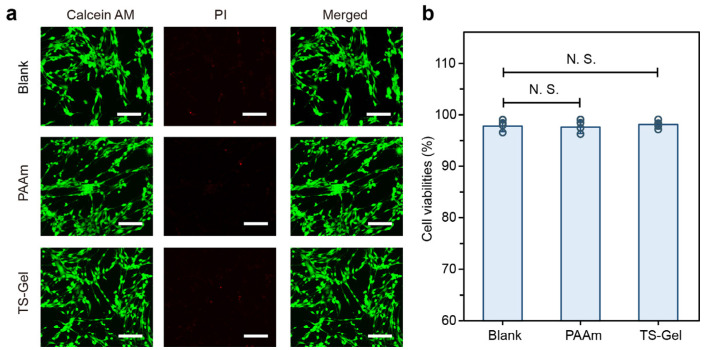
Cytocompatibility evaluation of TS-Gels. (**a**) Representative fluorescence images of live/dead stained cells cultured with blank, PAAm hydrogel, and TS-Gel after 72 h. Live cells are shown in green (Calcein AM) and dead cells in red (PI). Scale bars = 200 μm. (**b**) Quantification of cell viability measured using live/dead assays. Data are presented as mean ± standard deviation (*n* = 3). Statistical significance was evaluated using Student’s *t*-test. N.S.: not significant.

To evaluate the biomedical potential of the TS-Gels, we performed preliminary cytocompatibility assessments using standard live/dead viability assays. As shown in Figure 9a, fluorescence images of cells cultured with the blank control, PAAm hydrogels, and TS-Gels—stained with Calcein AM (live cells, green) and propidium iodide (dead cells, red)—revealed uniformly high cell viability across all the groups, with minimal red fluorescence, indicative of low cytotoxicity. No notable differences were observed in the cell morphology, density, or spreading among the groups, suggesting that the incorporation of tyrosine crystals and the ALP-mediated crystallization process did not impair cell attachment or viability.

Quantitative analysis further supported these findings. As depicted in Figure 9b, all the groups showed a cell viability consistently above 90%, with no statistically significant differences (N.S.) between the PAAm, TS-Gel, and blank control groups (*n* = 3). These results demonstrate that TS-Gels offer a cytocompatible environment suitable for supporting human cell growth, underscoring their potential for use in biomedical applications involving direct cell contact. No extracellular matrix coating was applied before MSC seeding, allowing us to assess the intrinsic cytocompatibility of the TS-Gel surfaces. While a period of 72 h was selected to capture potential late-onset cytotoxic effects and observe the cell attachment over time, we acknowledge that early assessments are important for detecting acute toxicity, and these will be included in future studies.

With their excellent mechanical tunability, programmable stiffness, and demonstrated cytocompatibility, TS-Gels present a promising platform for a wide range of biomedical applications. Their ability to form spatially patterned and mechanically anisotropic architectures enables the engineering of biomimetic tissue structures such as gradient cartilage–bone interfaces, ligament-to-tendon transitions, and organized neural scaffolds. In addition, the low-friction and wear-resistant properties of anisotropic TS-Gels make them attractive for use in bio-tribological interfaces, including artificial cartilage and in-body sensor components. Beyond their structural roles, the potential to induce localized mechanical patterning through light-initiated or laser engraving crystallization opens new avenues for the development of multifunctional hydrogel systems. These may include mechanoresponsive drug delivery devices, embedded hydrogel strain sensors, or programmable soft actuators that convert mechanical inputs into functional responses. In summary, the modularity, biocompatibility, and mechanical versatility of TS-Gels provide a robust and flexible platform for developing next-generation bio-integrated materials and devices.

## 3. Conclusions

We report a bioinspired strategy for constructing tensegrity-structured hydrogels (TS-Gels) by integrating enzymatically grown tyrosine crystal sticks within a prestretched PAAm network. Drawing from cytoskeletal design principles, this architecture enables the formation of a mechanically anisotropic and highly robust network through the spatial interplay of stiff crystalline elements and compliant polymer chains. The in situ crystallization process, governed by spatially distributed ALP activity or nucleation gradients, allows for programmable patterning of stiffness and mechanical anisotropy across length scales. Light-initiated printing and laser engraving crystallization further permit a submillimeter resolution in mechanical encoding. A bilayer TS-Gel architecture exhibiting both high lubricity and an enhanced load-bearing capacity demonstrates potential for use in low-friction and wear-resistant applications. Importantly, the TS-Gels exhibit excellent cytocompatibility, supporting their use in biomedical environments. These modular and versatile platforms provide a new material framework for encoding spatially heterogeneous mechanics into soft matter, with broad implications for tissue engineering and the development of adaptive biomaterials.

## 4. Materials and Methods

### 4.1. Materials

Acrylamide (AAm, 99%), N,N′-methylenebisacrylamide (BIS, 99%), L-tyrosine (98%), phosphotyrosine (95%), L-serine (99%), phosphoserine (98%), triethylamine (TEA, 99%), and alkaline phosphatase (ALP, ≥1.5 kU mg^−1^) were purchased from Sigma-Aldrich (St. Louis, MO, USA). Acrylated polypeptides bearing the SpyTag sequence (Acrylate-Ala-His-Ile-Val-Met-Val-Asp-Ala-Tyr-Lys-Pro-Thr-Lys, ≥95% purity) were obtained from GL Biochem (Shanghai, China). Lithium phenyl(2,4,6-trimethylbenzoyl) phosphinate (LAP, 98%) was sourced from TCI (Shanghai, China). Unless otherwise noted, 3-(trimethoxysilyl)propyl methacrylate (TMSPMA) and other reagents were purchased from Aladdin (Shanghai, China).

### 4.2. Protein Engineering

The gene encoding SpyCatcher–alkaline phosphatase (SpyCatcher–ALP) was custom-synthesized by GenScript (Nanjing, China) with codon optimization for expression in Escherichia coli. The construct included BamHI, BglII, and KpnI restriction sites at both termini. Individual gene fragments were ligated via BamHI/BglII overhangs and subcloned into the pET30a vector. All the constructs were verified by Sanger sequencing. Amino acid sequences are provided in Supplementary Table S1.

### 4.3. Preparation of Tyrosine Crystals and Nucleation Seeds

L-tyrosine was saturated in a 0.2 M TEA buffer (pH 8.8), followed by the addition of phosphotyrosine (38 mM). After a pH adjustment to 8.8, ALP (1 mg mL^−1^) was introduced to initiate enzymatic crystallization at 37 °C for 120 h. The resulting crystals were freeze-dried and ground using a TissueLyser (TL2020, DHS, Beijing, China) at 1000 rpm for 22.5 min to produce crystal seeds with a mean diameter of below 200 nm.

### 4.4. Atomic Force Microscopy (AFM)

AFM-based nanoindentation was conducted on a JPK Nanowizard IV system (Bruker, Billerica, MA, USA) using RTESPA-525 probes (Bruker, Billerica, MA, USA; ~200 N m^−1^ spring constant, ~10 nm tip radius). Dried crystal samples were deposited on mica substrates, and force–displacement curves were acquired in the QI mode over 5 × 5 μm^2^ areas. Young’s modulus was determined by fitting the results to the Hertz model.

### 4.5. Scanning Electron Microscopy (SEM)

Samples were freeze-dried and sputter-coated with a 15 nm gold layer. Imaging was performed using a GeminiSEM 360 (Zeiss, Oberkochen, Germany) at an accelerating voltage of 15 kV.

### 4.6. PAAm Hydrogel Preparation

Precursor solutions containing AAm (1000 mM), BIS (20 mM), acrylate–SpyTag (0.1 mM), SpyCatcher–ALP (0.05 mM), and LAP (0.85 mM) were prepared. Crystal nuclei (2 mg mL^−1^) were incorporated, and the mixture was incubated at room temperature for 10 min, then UV-polymerized (285 nm, 1 h) in custom molds. For tribological testing, the AAm, BIS, and LAP concentrations were adjusted to 4000 mM, 20 mM, and 2.0 mM, respectively. All the hydrogels were equilibrated in a saturated L-tyrosine solution until fully swollen. For all the experiments, the standard formulation contained crystal nuclei at 2 mg mL^−1^ and SpyCatcher–ALP at 0.05 mM. Preliminary tests indicated that reducing either component to approximately half of these amounts still produced measurable reinforcement, whereas contents of above ~10 wt% (for the crystals) or proportionally higher ALP concentrations were expected to compromise the uniformity of the hydrogel network.

### 4.7. In Situ Crystal Growth

Preformed PAAm hydrogels were immersed in a saturated L-tyrosine solution containing 50 mM phosphotyrosine (pH 8.8) and incubated at 37 °C for varying durations to induce crystal growth within the network. After the crystal growth, XRD patterns were recorded using a Bruker D8 Advance diffractometer at 25 °C (scanning speed: 0.5° s^−1^; 2θ range: 5–80°). FTIR spectra were collected on a NICOLET iS10 spectrometer (Thermo Fisher, Waltham, MA, USA) with ≥64 scans per sample and appropriate background subtraction to enhance the signal-to-noise ratio.

### 4.8. Crystal Mass Fraction Quantification

Hydrogels were weighed in their fully swollen state (*W*_1_) then freeze-dried to obtain their total dry mass (*W*_2_). The polymer dry mass (*W*_0_) was estimated from the initial composition. The crystal mass fraction was defined as *W*_t_/*W*_120_ × 100%, where *W*_t_ represents the crystal mass after *t* hours of incubation, and *W*_120_ corresponds to the mass after 120 h.

### 4.9. Light-Initiated Printing of TS-Gels

A precursor solution containing AAm (1000 mM), BIS (20 mM), acrylate–SpyTag (0.1 mM), SpyCatcher–ALP (0.05 mM), and LAP (0.85 mM) in a saturated L-tyrosine solution was incubated at room temperature for 10 min to allow for SpyTag–SpyCatcher coupling. After incorporation of crystal nuclei (2 mg mL^−1^), the ink was printed using a DLP 3D printer (CUV4704, Crystal-Optech, Taizhou, China). The printed structures were incubated at 37 °C for 1 h to facilitate in situ crystallization.

### 4.10. Mechanical Testing

The tensile and compressive properties were assessed using an Instron 5944 universal tester (2 kN load cell). Tensile specimens (14 × 8 × 1.8 mm^3^) and cylindrical compression samples (Φ7 × 4.5 mm) were tested at 1 mm min^−1^ under uniaxial loading.

### 4.11. Fabrication of Bilayer Hydrogels (bTS-Gels)

To ensure robust adhesion between the PAAm and TS-Gel layers, 10 mM TMSPMA was added to both precursor solutions. TMSPMA-derived Si–OH groups promoted covalent crosslinking via dehydration–rehydration using ethanol and water, enabling stable hydrogel–hydrogel and hydrogel–substrate integration.

### 4.12. Biocompatibility Assessment

Human bone marrow-derived mesenchymal stem cells (hMSCs) were cultured in DMEM with 10% fetal bovine serum and 1% penicillin–streptomycin at 37 °C and 5% CO_2_. Fourth-passage cells (1 × 10^4^ cells/well) were seeded on sterilized PAAm or TS-Gel disks (Φ3 mm × 1.8 mm) in 24-well plates and incubated for 72 h (*n* = 6). A cell-only blank served as a control. The cell viability was evaluated using Calcein AM/PI staining. After rinsing the samples with PBS, the samples were stained with Calcein AM and PI (both 1:500) at 37 °C for 30 min, rinsed again, and imaged by confocal microscopy (Olympus FV3000, Tokyo, Japan). Live/dead quantification was performed using MATLAB R2024b based on thresholding and watershed segmentation (see Supplementary Table S3).

## Data Availability

The data presented in this study are available on request from the corresponding author.

## Supplementary Materials

The following supporting information can be downloaded at: https://www.mdpi.com/article/10.3390/gels11080654/s1, Figure S1: Cytoskeletal model and tensegrity concept in hydrogels; Figure S2: ALP-induced tyrosine crystallization forms tensegrity networks; Figure S3: SEM of tyrosine crystal nuclei; Figure S4: FTIR and XRD of crystals and hydrogels; Figure S5: DSC and DTA of PAAm and TS-Gels; Figure S6: AFM morphology and modulus of tyrosine crystals; Figure S7: Morphology and mechanics of TS-Gels and PAAm; Figure S8: Time-lapse crystal growth in TS-Gels; Figure S9: Workflow for crystal growth image analysis; Figure S10: Modulus mapping by crystal nucleation pattern; Figure S11: Modulus mapping by ALP distribution; Table S1: Amino acid sequence of proteins used in this study; Table S2: MATLAB code of the Tyrosine crystal recognition algorithm; Table S3: MATLAB code for quantifying live and dead cell populations.
