# Supplementary material for: Enzyme-Triggered Formation of Tensegrity Structures for Mechanospatial Manipulation of Hydrogels
Source: Gels. 2025 Aug 18;11(8):654. doi: 10.3390/gels11080654 (PMC12385324; doi:10.3390/gels11080654)
Supplement: Supplementary file 1 [file gels-11-00654-s001.zip › gels-3797696-supplementary.pdf]

Supplementary Materials

Enzyme-Triggered Formation of Tensegrity Structures for Mechanospatial Manipulation of Hydrogels

Juan Wang [1,2], Xu Han [1], Qingtai Li [1], Meng Qin [1], Bin Xue [1,2], Wenxu Sun [1,3,*], Yi Cao [1,2,4] and Wei Sun [1,*]

[1] Collaborative Innovation Center of Advanced Microstructures, National Laboratory of Solid State
Microstructure, Department of Physics, Nanjing University, Nanjing 210008, China; wangjuannm@163.com (J.W.); dz20220007@smail.nju.edu.cn (X.H.); lqt861563391@163.com (Q.L.); qinmeng@nju.edu.cn (M.Q.); xuebinnju@nju.edu.cn (B.X.); caoyi@nju.edu.cn (Y.C.)

[2] Jinan Microecological Biomedicine Shandong Laboratory, Jinan 250000, China

[3] School of Physical Science and Technology, Nantong University, Nantong 226019, China

[4] Chemistry and Biomedicine Innovation Center, School of Chemistry and Chemical Engineering, Nanjing University, Nanjing 210000, China

* Correspondence: sunwenxu@ntu.edu.cn (W.S.); sunwei@nju.edu.cn (W.S.)

This PDF file includes:

Supplementary Figures 1 to 11

Supplementary Tables 1 to 3

Supplementary Figures

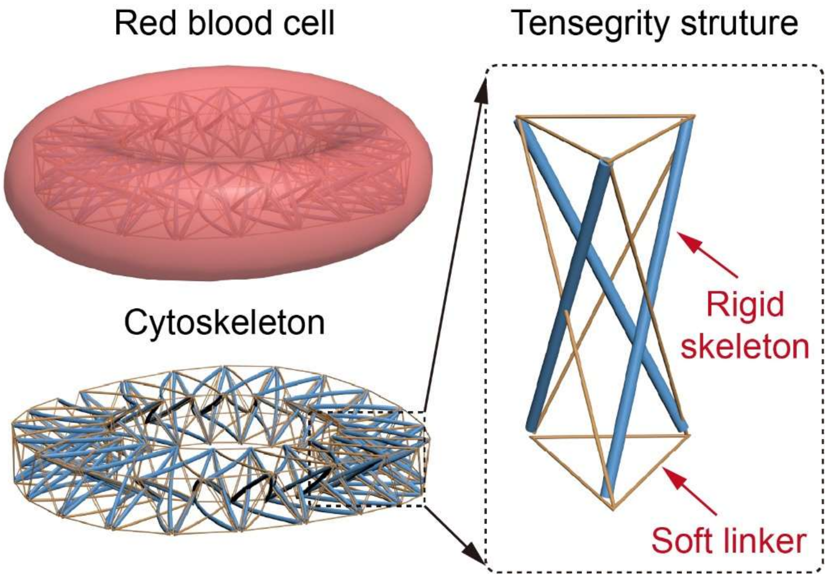

Supplementary Figure 1. Illustration of the cytoskeletal architecture in red blood cells formed by microtubules and actin filaments (top), and a conceptual tensegrity structure composed of rigid tyrosine crystal sticks and flexible polymer chains (bottom).

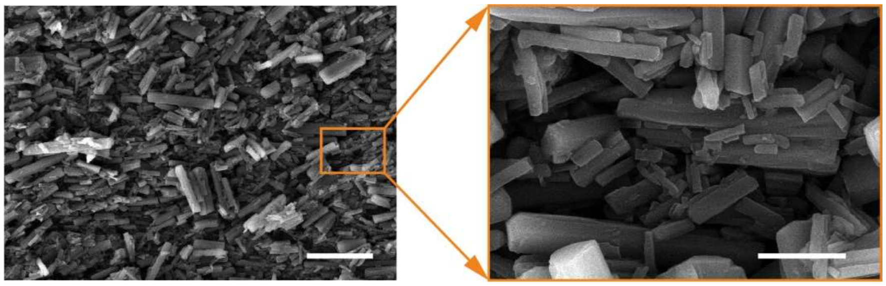

Supplementary Figure 3. SEM images of tyrosine crystal nuclei. Scale bars: 40 μm (right) and 5 μm (left).

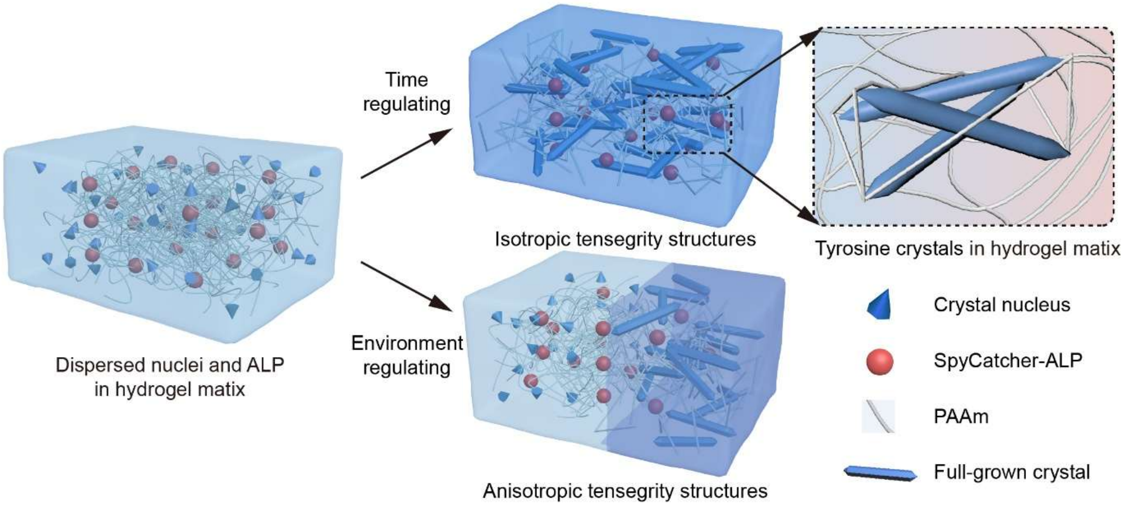

Supplementary Figure 2. Formation of tensegrity structures in the hydrogel through ALP-induced in situ crystallization. ALP is covalently incorporated into the hydrogel matrix to trigger localized crystal growth. The spatial mechanical properties of the hydrogel can be precisely tuned by controlling crystal growth time, the distribution of nucleation sites, and ALP concentration.

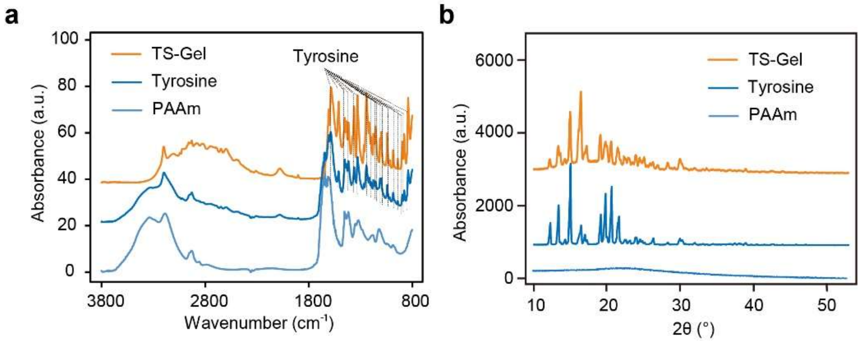

Supplementary Figure 4. (a) FTIR spectra and **(b)** XRD patterns of powdered tyrosine crystals, TS-Gel, and PAAm hydrogels.

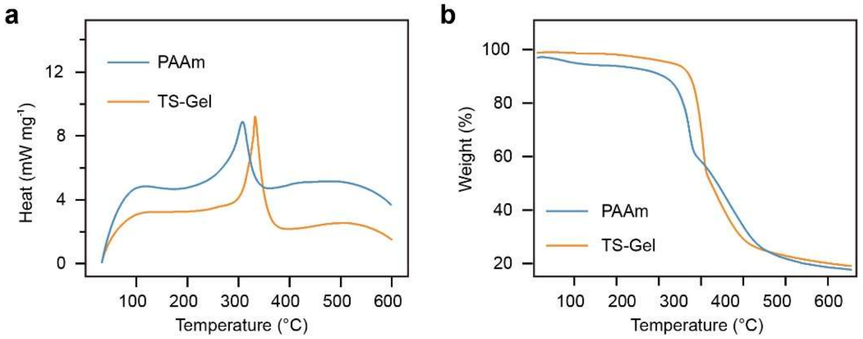

Supplementary Figure 5. (a) Differential scanning calorimetry (DSC) and **(b)** differential thermal analysis (DTA) of PAAm and TS-Gel hydrogels.

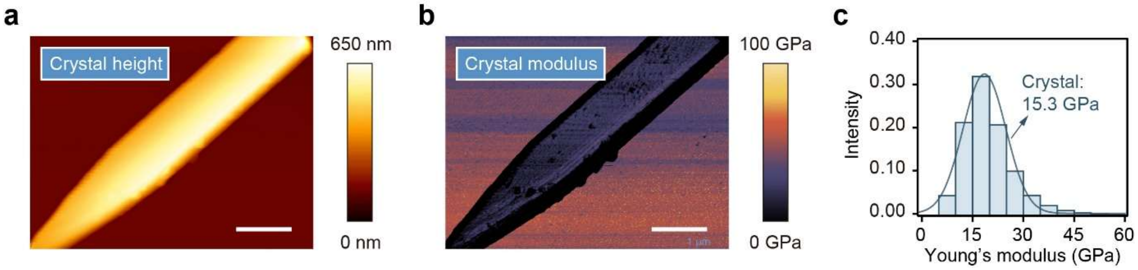

Supplementary Figure 6. Characterization of tyrosine crystals formed via enzyme-triggered crystallization. **(a)** Representative AFM height image and **(b)** corresponding modulus map of tyrosine crystals obtained by nanoindentation. Scale bars: 1 μm. **(c)** Statistical distribution of Young's modulus for tyrosine crystals, averaged from five QI-mode AFM images (5 μm × 5 μm).

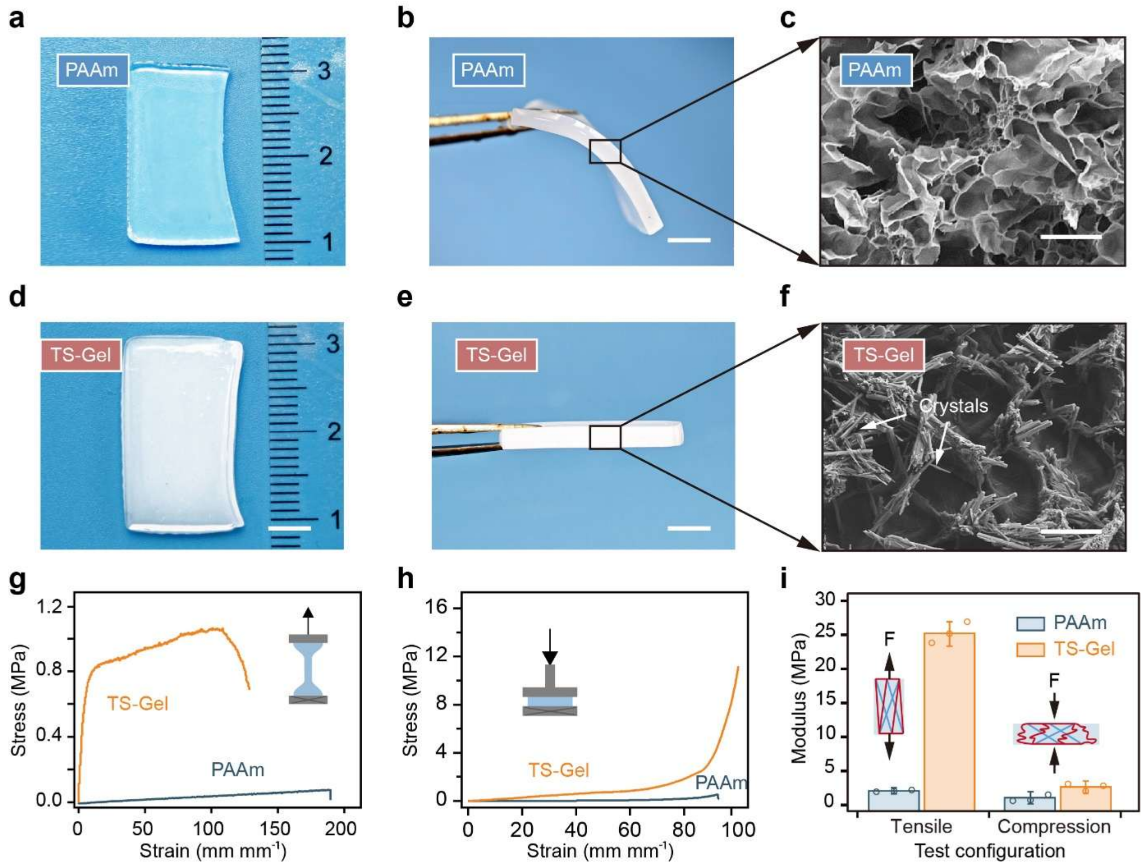

Supplementary Figure 7. Characterization of TS-Gels and PAAm hydrogels. **(a)** Front-view optical, **(b)** side-view optical, and **(c)** SEM images of PAAm hydrogels. Scale bars: 5 mm (optical) and 40 μm (SEM). **(d)** Front-view optical, **(e)** side-view optical, and **(f)** SEM images of TS-Gels. Scale bars: 5 mm (optical) and 40 μm (SEM). **(g)** Representative tensile and **(h)** compressive stress–strain curves of TS-Gels and PAAm hydrogels. **(i)** Comparison of Young's modulus values for TS-Gels and PAAm hydrogels under tensile and compressive loading. Data are presented as mean ± standard deviation (n = 3).

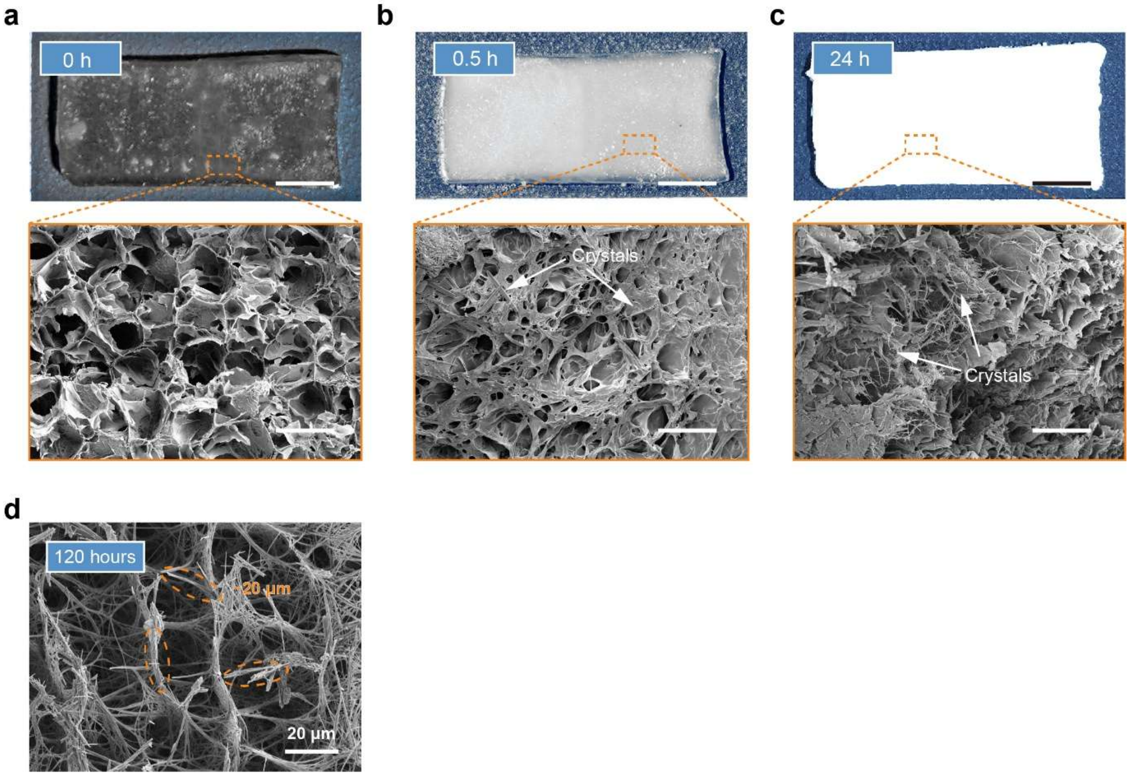

Supplementary Figure 8. Evolution of tyrosine crystal growth within the TS-Gel polymer network. Optical (top) and SEM (bottom) images of TS-Gels after in situ crystallization for **(a)** 0 hours, **(b)** 0.5 hours, **(c)** 24 hours, and **(d)** 120 hours. The optical images illustrate the macroscopic transition of the hydrogel from translucent to opaque, while the SEM images reveal the emergence and spatial distribution of crystal sticks embedded within the polymer matrix. Scale bars: 5 mm (optical) and 100 µm (SEM).

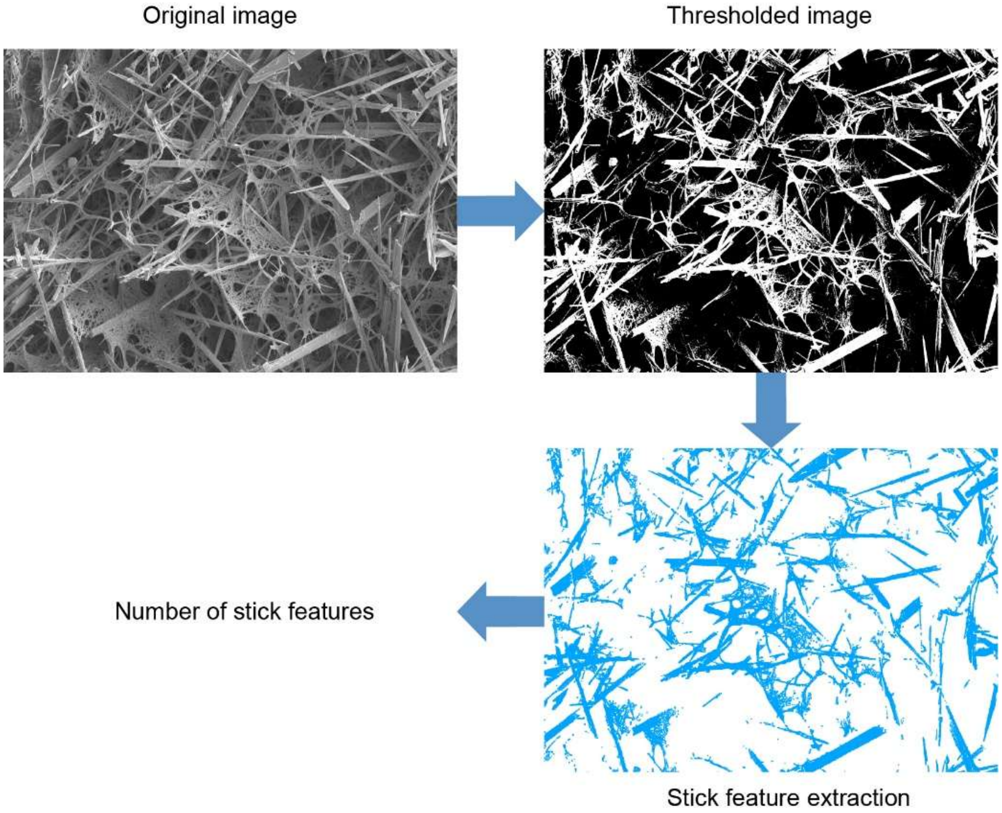

Supplementary Figure 9. Image processing workflow used to analyze the growth kinetics of tensegrity structures.

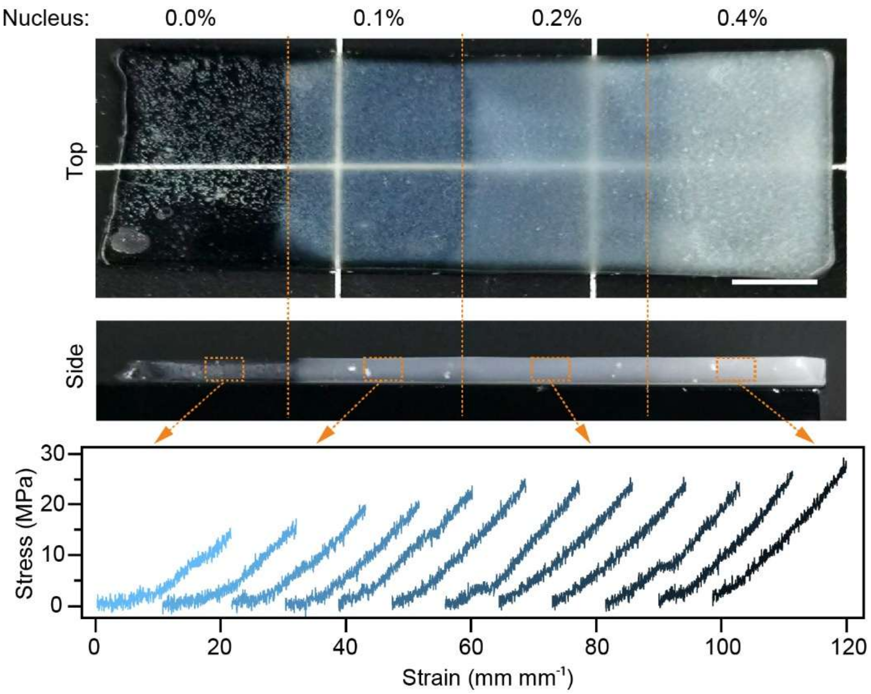

Supplementary Figure 10. Spatial heterogeneity of tensile modulus in hydrogels regulated by the distribution of crystal nuclei. Hydrogels with spatially patterned crystal nucleation sites exhibited region-specific mechanical properties. Representative tensile tests revealed that regions with higher nucleation densities showed significantly increased modulus compared to regions without nucleation. Scale bar: 2 mm.

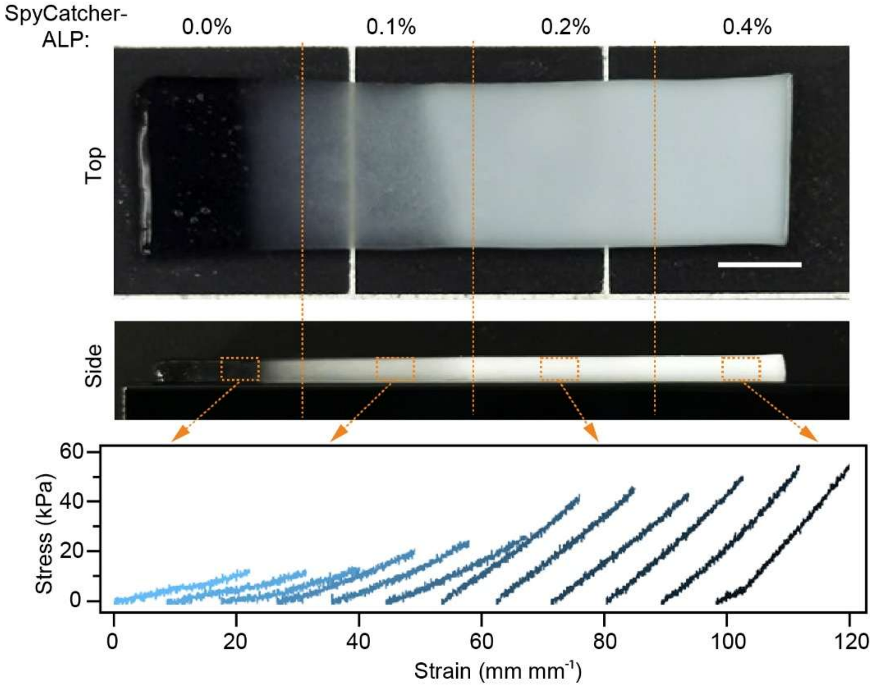

Supplementary Figure 11. Spatial heterogeneity of tensile modulus in hydrogels regulated by ALP distribution. Hydrogels with spatially patterned ALP concentrations displayed distinct regional mechanical properties. Areas with higher ALP levels promoted greater crystal growth, resulting in locally enhanced stiffness. Scale bar: 2 mm.

Supplementary Tables

Supplementary Table 1. Amino acid sequence of proteins used in this study.

SC-ALP
HHHHHHGSAMVTTLSGLSGEQGPSGDMTTEEDSATHIKFSKRDEDGRELAGATMELRDSSGKTISTWISDGHVKDFYLYPGKY
TFVETAAPDGYEVATPIEFTVNEDGQVTVDGEATEGDAHTRSMPVLENRAAQGDITAPGGARRLTGDQTAALRDSLSDKPAK
NIILLIGDGMGDSEITAARNYAEGAGGFFKGIDALPLTGQYTHYALNKKTGKPDYVTDSAASATAWSTGVKTYNGALGVDIHE
KDHPTILEMAKAAGLATGNVSTAELQDATPAALVAHVTSRKCYGPSATSEKCPGNALEKGGKGSITEQLLNARADVTLGGGA
KTFAETATAGEWQGKTLREQAQARGYQLVSDAASLNSVTEANQQKPLLGLFADGNMPVRWLGPKATYHGNIDKPAVTCTPN
PQRNDSVPTLAQMTDKAIELLSKNEKGFFLQVEGASIDHQDHAANPCGQIGETVDLDEAVQRALEFAKKEGNTLVIVTADHA
HASQIVPDTKAPGLTQALNTKDGAVMVMSYGNSEEDSQEHTGSQLRIAAYGPHAANVVGLTDQTDLFYTMKAALGLK

Supplementary Table 2. MATLAB code of the Tyrosine crystal recognition algorithm used in this study.

Tyrosine crystal recognition algorithm
% 1. Read the image and preprocess<br>img = imread('data.png'); % Replace with your image path<br>gray_img = rgb2gray(img); % Convert to grayscale if RGB<br>bin_img = imbinarize(gray_img); % Binarization (default threshold)<br><br>% 2. Identify white fiber regions (black background with white fibers)<br>bin_img = imcomplement(bin_img); % Invert: white fibers become foreground (1)<br><br>% 3. Optimize fiber identification (morphological processing)<br>bin_img = bwareaopen(bin_img, 50); % Remove small noise [2,6](@ref)<br>bin_img = imclose(bin_img, strel('disk', 3)); % Connect broken fibers [5](@ref)<br><br>% 4. Connected component analysis (fiber counting)<br>[L, num_fibers] = bwlabel(bin_img, 8); % 8-connectivity region labeling [7,8](@ref)

```
fprintf('Detected fiber count: %d\n', num_fibers);

% 5. Extract fiber features (optional)
fiber_stats = regionprops(L, 'Area', 'Centroid'); % Get area and centroid [7](@ref)
fiber_areas = [fiber_stats.Area]; % Array of all fiber areas

% 6. Create a blue highlight effect
blue_highlight = img; % Preserve original image structure
blue_highlight(repmat(bin_img, [1,1,3])) = 0; % Clear original color (set R/G channels to 0)
blue_highlight(:,:,3) = blue_highlight(:,:,3) + uint8(bin_img)*255; % Enhance blue channel

% 7. Annotate fiber count on the image
result_img = insertText(blue_highlight, [10,10], ...
    sprintf('Fibers: %d', num_fibers), ...
    'FontSize', 20, 'BoxColor','black','TextColor','white'); % English annotation [6,7](@ref)
```

```
% 8. Display and save results
figure;
subplot(1,2,1), imshow(img), title('Original Image');
subplot(1,2,2), imshow(result_img), title(['Fiber Count: ',num2str(num_fibers)]);
imwrite(result_img, 'fiber_result.png');
```

Supplementary Table 3. MATLAB code for quantifying live and dead cell populations used in this study.

Cell populations algorithm
% 1. Read a color image<br>img = imread('data.tif'); % Replace with your tif image path<br><br>% 2. Convert to a grayscale image<br>gray_img = rgb2gray(img); % Convert RGB image to grayscale<br><br>% 3. Binarize image (automatic thresholding)<br>% Use Otsu's method to automatically calculate optimal threshold<br>threshold = graythresh(gray_img); % Calculate binarization threshold<br>binary_img = imbinarize(gray_img, threshold); % Apply threshold for binarization<br><br>% 4. Noise removal (morphological processing)<br>% Perform opening operation to eliminate small particle noise<br>se = strel('disk', 10); % Create circular structuring element with radius 10

```
clean_img = imopen(binary_img, se); % Execute opening operation (erosion followed by dilation)

% 5. Identify connected components
cc = bwconncomp(clean_img); % Detect connected pixel regions in the image

% 6. Cell counting
num_cells = cc.NumObjects; % Count connected regions as cell quantity

% Display result
fprintf('Detected cell count: %d\n', num_cells); % Output cell count

% (Optional) Visualization
% imshow(clean_img); % Display processed binary image
% title(['Cell count: ', num2str(num_cells)]); % Display count on image
```
